# Supplementary material for: HIVprotI: an integrated web based platform for prediction and design of HIV proteins inhibitors
Source: J Cheminform. 2018 Mar 9;10:12. doi: 10.1186/s13321-018-0266-y (PMC5845081; doi:10.1186/s13321-018-0266-y)
Supplement: Supplementary file 2 — Additional file 2. Source code of HIVProtI web server. [file 13321_2018_266_MOESM2_ESM.zip › HIVprotI_Source-code/hivprotI/weka-3-6-6/doc/weka/gui/beans/TrainingSetEvent.html]

TrainingSetEvent


---


|  |  |  |
| --- | --- | --- |
| **Overview** \| **Package** \| **Class** \| **Tree** \| **Deprecated** \| **Index** \| **Help** \| **Weka's home** | |  |
| **PREV CLASS**   **NEXT CLASS** | **FRAMES**    **NO FRAMES**     **All Classes** |  |
| SUMMARY: NESTED \| FIELD \| CONSTR \| METHOD | DETAIL: FIELD \| CONSTR \| METHOD |  |


---


## weka.gui.beans Class TrainingSetEvent

```
java.lang.Object
  java.util.EventObject
      weka.gui.beans.TrainingSetEvent
```

**All Implemented Interfaces:**: java.io.Serializable

---

``` public class TrainingSetEvent extends java.util.EventObject ```

Event encapsulating a training set

**Version:**
:   $Revision: 4761 $

**Author:**
:   Mark Hall

**See Also:**: Serialized Form

---

**Constructor Summary**

`TrainingSetEvent(java.lang.Object source,
Instances trainSet)`
  
          Creates a new `TrainingSetEvent`

`TrainingSetEvent(java.lang.Object source,
Instances trainSet,
int setNum,
int maxSetNum)`
  
          Creates a new `TrainingSetEvent`

`TrainingSetEvent(java.lang.Object source,
Instances trainSet,
int runNum,
int maxRunNum,
int setNum,
int maxSetNum)`
  
          Creates a new `TrainingSetEvent`


| **Method Summary** | |
| --- | --- |
| `int` | `getMaxRunNumber()`             Get the maximum number of runs. |
| `int` | `getMaxSetNumber()`             Get the maximum set number |
| `int` | `getRunNumber()`             Get the run number that this training set belongs to. |
| `int` | `getSetNumber()`             Get the set number (eg. |
| `Instances` | `getTrainingSet()`             Get the training instances |
| `boolean` | `isStructureOnly()`             Returns true if the encapsulated instances contain just header information |

**Methods inherited from class java.util.EventObject**

`getSource, toString`

**Methods inherited from class java.lang.Object**

`equals, getClass, hashCode, notify, notifyAll, wait, wait, wait`

**Constructor Detail**

### TrainingSetEvent

```
public TrainingSetEvent(java.lang.Object source,
                        Instances trainSet)
```

:   Creates a new `TrainingSetEvent`

    **Parameters:**: `source` - the source of the event: `trainSet` - the training instances

---


### TrainingSetEvent

```
public TrainingSetEvent(java.lang.Object source,
                        Instances trainSet,
                        int setNum,
                        int maxSetNum)
```

:   Creates a new `TrainingSetEvent`

    **Parameters:**: `source` - the source of the event: `trainSet` - the training instances: `setNum` - the number of the training set: `maxSetNum` - the maximum number of sets

---


### TrainingSetEvent

```
public TrainingSetEvent(java.lang.Object source,
                        Instances trainSet,
                        int runNum,
                        int maxRunNum,
                        int setNum,
                        int maxSetNum)
```

:   Creates a new `TrainingSetEvent`

    **Parameters:**: `source` - the source of the event: `trainSet` - the training instances: `runNum` - the run number that the training set belongs to: `maxRunNum` - the maximum run number: `setNum` - the number of the training set: `maxSetNum` - the maximum number of sets


**Method Detail**

### getTrainingSet

```
public Instances getTrainingSet()
```

:   Get the training instances

    :   **Returns:**: an `Instances` value

---


### getRunNumber

```
public int getRunNumber()
```

:   Get the run number that this training set belongs to.

    :   **Returns:**: the run number for this training set.

---


### getMaxRunNumber

```
public int getMaxRunNumber()
```

:   Get the maximum number of runs.

    :   **Returns:**: return the maximum number of runs.

---


### getSetNumber

```
public int getSetNumber()
```

:   Get the set number (eg. fold 2 of a 10 fold split)

    :   **Returns:**: an `int` value

---


### getMaxSetNumber

```
public int getMaxSetNumber()
```

:   Get the maximum set number

    :   **Returns:**: an `int` value

---


### isStructureOnly

```
public boolean isStructureOnly()
```

:   Returns true if the encapsulated instances
    contain just header information

    :   **Returns:**: true if only header information is available in this DataSetEvent


---


|  |  |  |
| --- | --- | --- |
| **Overview** \| **Package** \| **Class** \| **Tree** \| **Deprecated** \| **Index** \| **Help** \| **Weka's home** | |  |
| **PREV CLASS**   **NEXT CLASS** | **FRAMES**    **NO FRAMES**     **All Classes** |  |
| SUMMARY: NESTED \| FIELD \| CONSTR \| METHOD | DETAIL: FIELD \| CONSTR \| METHOD |  |


---
